# CSF α‐synuclein reflects neurodegeneration in Alzhiemer's disease

**DOI:** 10.1002/alz.083793

**Published:** 2025-01-09

**Authors:** Kazuya Igarashi, Kensaku Kasuga, Tamao Tsukie, Masataka Kikuchi, Akinori Miyashita, Takeshi Iwatsubo, Takeshi Ikeuchi

**Affiliations:** ^1^ Department of Molecular Genetics, Brain Research Institute, Niigata University, Niigata Japan; ^2^ Department of Molecular Genetics, Brain Research Institute, Niigata University, Niigata, Niigata Japan; ^3^ Department of Computational Biology and Medical Sciences, Graduate School of Frontier Science, The University of Tokyo, Chiba Japan; ^4^ Brain Research Institute, Niigata University, Niigata, Niigata Japan; ^5^ Department of Neuropathology, Graduate School of Medicine, University of Tokyo, and the National Center of Neurology and Psychiatry, Tokyo Japan

## Abstract

**Background:**

The aim of this study is to investigate the clinical significance of cerebrospinal fluid (CSF) α‐synuclein (αSyn), a presynaptic protein, in Alzheimer's disease (AD) continuum.

**Method:**

We analyzed 177 CSF samples obtained from participants of Japanese Alzheimer’s Disease Neuroimaging Initiative (J‐ADNI). To investigate the clinical utility of CSF αSyn, subjects were divided into cognitively unimpaired (CU) group (n = 46), mild cognitive impairment (MCI) group (n = 76), and AD dementia (ADD) group (n = 43). We measured CSF αSyn, amyloid β (Aβ)42, phosphorylated tau (pTau), total tau (tTau), Neurofilament Light chain (NfL), hemoglobin (Hb). Because high CSF Hb levels affect αSyn levels due to red blood cell contamination, 12 patients with CSF Hb levels of 1000 ng/mL or higher were excluded. We analyzed the correlation between CSF αSyn and age, gender differences, cognitive function, brain volume (entorhinal cortex, hippocampus, ventricle, cortex, white matter, gray matter, white matter hyperintensities), and each biomarker.

**Result:**

CSF αSyn were significantly higher in the MCI and ADD groups than in the CU group, and significantly negatively correlated with the MMSE score. The hazard ratio for conversion to dementia was significantly higher in the CSF αSyn high and middle tertile groups compared to the low tertile group. CSF αSyn was also associated with baseline and 24‐month score change of MMSE, ADAS, CDR‐SB, and FAQ. CSF αSyn were significantly positively correlated with CSF pTau181, tTau, and NfL and hippocampal volume, but not with CSF Aβ42.

**Conclusion:**

Our results suggests CSF αSyn is a biomarker reflecting neurodegeneration in the Alzheimer's continuum.

